# A Review of Bark-Extract-Mediated Green Synthesis of Metallic Nanoparticles and Their Applications

**DOI:** 10.3390/molecules24234354

**Published:** 2019-11-28

**Authors:** Ema Burlacu, Corneliu Tanase, Năstaca-Alina Coman, Lavinia Berta

**Affiliations:** 1 Residency Department, “George Emil Palade” University of Medicine, Pharmacy, Sciences and Technology of Târgu Mureș, 38 Gheorghe Marinescu Street, Târgu Mureș, 540139 Mureș, Romania; morariuemma@yahoo.com; 2 Department of Pharmaceutical Botany, “George Emil Palade” University of Medicine, Pharmacy, Sciences and Technology of Târgu Mureș, 38 Gheorghe Marinescu Street, Târgu Mureș, 540139 Mureș, Romania; alinacoman2194@yahoo.com; 3 Department of General and Inorganic Chemistry, “George Emil Palade” University of Medicine, Pharmacy, Sciences and Technology of Târgu Mureș, 38 Gheorghe Marinescu Street, Târgu Mureș, 540139 Mureș, Romania; lavinia.berta@umfst.ro

**Keywords:** metallic nanoparticles, bark extract, woody vascular plants, phytoconstituents, antioxidant, antimicrobial, anticancer

## Abstract

Nanoparticles are intensely studied because of their importance in diverse fields of biotechnology, especially in medicine. This paper highlights that waste bark can be a cheap source of biocompounds, with high recovery and functionalization potential in nanoparticle synthesis. Due to their biocompatibility and activity as antioxidant, antimicrobial, and anticancer agents, the green synthesis of metallic nanoparticles is of great importance. This review aims to bring together the diversity of synthesized metallic nanoparticles mediated by bark extracts obtained from different woody vascular plants, the phytoconstituents responsible for the reduction of metal salts, and the activity of metallic nanoparticles as diverse agents in combating the microbial, oxidant, and cancer activity. The literature data highlight the fact that metallic nanoparticles obtained from natural compounds are proven reducing agents with multiple activities. Thus, the activity of natural components in environmental protection and human health is confirmed.

## 1. Introduction

In recent years, nanobiotechnology has attracted considerable research, which has had an impact on all life forms [1]. Nanoparticles (NPs) have shown notable advances owing to a wide range of applications in biomedicine, sensors, antimicrobial agents, catalysts, electronics, optical fibers, agricultural, biolabeling, and other areas [2]. Due to their particle size, various shapes, and increased surface area, nanoparticles present very different properties than their bulk materials. The interest in NPs’ applicability in different fields, especially in biomedical science, is increasing with every new research study in this domain [3].

NPs can be prepared and stabilized by physical and chemical methods. Chemicals used in chemical synthesis are toxic and lead to non-ecofriendly by-products. This is the reason for the need for green synthesis of NPs without employing toxic chemicals [4]. However, with the rapid development of the timber industry, the use of biological methods to protect the environment has been proposed. An advancement in green synthesis of NPs was the use of biological entities such as microorganisms or plant extracts for the production of NPs in an eco-friendly manner [5,6]. The use of plant extracts in the production assembly of metallic NPs is rapid, eco-friendly, non-pathogenic, and economical. The reduction and stabilization of metallic ions can be achieved with a combination of biomolecules with medicinal values, such as enzymes, polysaccharides, tannins, phenolics, saponins, terpenoids, among others [4].

**Figure 1:**
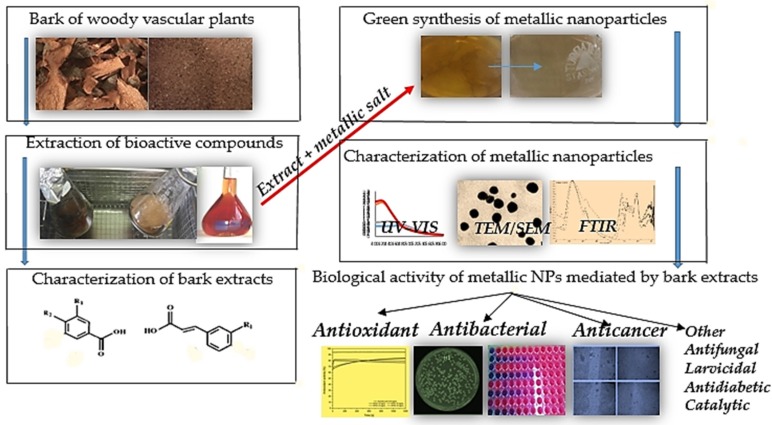
Green synthesis and biological activity of metallic nanoparticles mediated by bark extracts.

The green synthesis of nanoparticles has been improved in order to design new materials that are ecological, valuable, and stable [5,6]. In this context, bark extracts have excellent properties as bioreductants. This is mainly due to their content of phenolic compounds, from which NPs can be synthesized with controlled size and shape, greater stability, and more biocompatibility [7,8]. Thus, a work plan for obtaining nanoparticles mediated by bark extracts, their characterization, and their applicability was developed (Figure 1).

The aim of this review is to summarize the literature data (2012–2019) on metallic nanoparticles mediated by plant bark extracts, the nanoparticles’ characteristics, and to discuss their properties, including anticancer, antimicrobial, antioxidant, and other activities. The literature data used in this paper was collected via PubMed and Google Academic (2012–2019). The search terms were: metallic nanoparticles, bark extract, woody vascular plants, phytoconstituents, reducing agents, antioxidants, antimicrobial, and anticancer.

## 2. The Bark of Woody Vascular Plants—A Source of Phytoconstituents Responsible for Reduction of Metallic Ions in Nanoparticle Synthesis

In the timber industry, bark is considered a waste product used for combustion and as garden mulch. Some studies have developed a different process to remove valuable biocompounds from bark in order to produce high-tech, valuable materials [9,10,11]. The bark of woody vascular plants play important roles in plant protection because of their content of bioactive compounds with antimicrobial effects [12]. In a recent study, it was found that bark extracts may be a good source of reducing agents in synthesized metallic NPs [9].

Studies cited in this review established that regarding the composition of the majority of plant bark extracts, the bioactive compounds are polyphenols, alkaloids, terpenoids, carbohydrates, proteins, saponins, and vitamins (Table 1).

**Figure 2:**
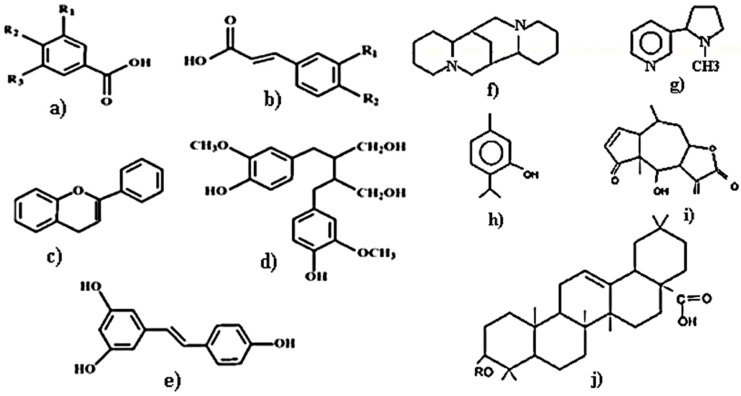
General structures of bioactive compounds identified in the bark extracts: (**a**) benzoic phenolic acids; (**b**) cinnamic phenolic acids; (**c**) flavonoids; (**d**) lignans; (**e**) stilbenes; (**f**,**g**) alkaloids; (**h**) monoterpenes; (**i**) sesquiterpenes; (**j**) triterpenes and saponins.

The polyphenols (Figure 2) are divided into sub-groups, such as phenolic acids (benzoic acid and cinnamic acid), flavonoids (flavan-3-ols, flavones, flavanones, and flavonols), anthocyanins, tannins, stilbenes (resveratrol), and lignans [13]. Majumdar et al. [14] showed the presence of phenolic compounds in the bark extract of *Mimusops elengi* L. (Sapotaceae) from a positive ferric chloride test. The phenolic compounds present in the bark extract of the plant can reduce Au (III) to Au (0), with concomitant oxidation of the phenolic compounds to the corresponding quinones. The stabilization of the gold nanoparticles (AuNPs) is done by the resulting quinone derivatives and other coordinating ligands present in the bark extract.

Terpenes (Figure 2) represent the biggest group of bioactive compounds found in plants, including monoterpenes (carvone, geraniol, d-limonene, peril alcohol), diterpenes (retinol and retinoic acid), triterpenes (boswellic acid, betulinic acid, lupeol, oleanolic acid, and ursolic acid), and tetraterpene (α-carotene, β-carotene, lutein, and lycopene). It has been found that this class of phytoconstituents act as a biological antioxidants [15,16].

For example, stem bark extract from *Terminalia arjuna* Wight and Arn (Combretaceae) showed phytoconstituents such as polyphenols, flavonoids, terpenoids, and reducing sugars [17]. The term reducing sugars refers to monosaccharides (glucose and fructose), along with some disaccharides, oligosaccharides, and polysaccharides [18]. Santos et al. [19] concluded that the reduction power is higher when the reducing sugars and phenolic compounds are present together in the plant extract. To better understand these compounds’ involvement, a mixed solution of pure glucose, fructose, gallic and ellagic acids, and isorhamnetin was tested, and the prompt formation of silver nanoparticles (AgNPs) and gold nanoparticles (AuNPs) was observed. The NPs synthesized were more stable than those formed by an aqueous solution of polyphenols only.

In another study, it was shown that the *Acacia leucophloea* Roxb. (Fabaceae) stem bark extract contained aldehyde/ketone, aromatic, azo, and nitro compounds. These compounds act as reducing and stabilizing agents of the AgNPs. Interestingly, plants have the incredible ability to enclose nanoparticles, forming a membrane around them and stopping the nanoparticles from forming aggregates, and in doing so stabilize the solution [20].

Moyo et al. [21] reported that *Afzelia quanzensis* Welw. (Fabaceae) bark extract contains phytochemical functional groups (carboxyl, amine). Such functional groups that are specific for polyphenols and proteins are confirmed reducing agents for nanoparticle formation.

The role of the proteins as reducing agents is still being discussed, and in the context, Santos et al. [19] decided to verify the content of proteins in the bark extract of *Eucalyptus globulus* Labill. (Myrtiaceae) and the role of proteins in nanoparticle reduction. Through elemental analysis they found that the protein content of the *E. globulus* bark extract represented approximately 0.8 wt% of the total extract, which corresponded to a concentration of approximately 19.0 mg/mL of the aqueous extract. They affirmed that proteins with a low concentration could not participate in the nanoparticle synthesis. However, proteins have great importance when it comes to the stabilization of NPs due to the affinity of binding metals with the carbonyl group of amino acid residues of proteins [22].

Miri et al. [23] suggested that various sizes of AgNPs can be related to diverse reducing factors present in different parts of the plant, such as tannins, flavonoids, enzymes, and alkaloids. Some examples of alkaloids are well-known in the medical world and are represented by atropine, codeine, morphine, and nicotine [24].

Saponins are less common as reducing agents in metallic nanoparticle formation, but in a previous paper [25] these phytoconstituents were isolated from *Trianthema decandra* L. and obtained rapidly stable silver and gold nanoparticles.

The clear mechanism and the phytoconstituents responsible for metallic ion reduction in nanoparticle synthesis remains to be elucidated. It has been proposed (Table 1) that flavonoids, alkaloids, polyphenols, terpenoids, heterocyclic compounds, and polysaccharides have significant roles in metal salt reduction, and furthermore, act as capping and stabilizing agents for NP synthesis.

## 3. Characteristics of Metallic Nanoparticles Mediated by the Bark Extracts of Woody Plants

Silver nanoparticles mediated by bark extracts revealed dimensions of 10–100 nm and spherical shapes with face-centered-cubic (FCC) structures in almost all of the variants (Table 1). For example, the AgNPs mediated by *Moringa oleifera* Lam. (Moringaceae) bark extract revealed a spherical-pentagonal shape [26]. A spherical-oval shape was characteristic for AgNPs mediated by *Toxicodendron vernicifluum* (Stokes) F. Barkley bark extract [27].

Gold nanoparticles have dimensions of 3–98 nm (Table 1). The shape of the obtained nanoparticles is generally spherical. For example, the shapes of NPs reduced by *Saraca indica* L. (Fabaceae) bark extract were triangular, tetragonal, pentagonal, hexagonal, and spherical [28]. The ellipsoidal shape was characteristic for NPs extracted with *Syzygium jambos* (L.) Alston (Myrtaceae) stem bark extract [29] and a triangular shape was observed in AuNPs obtained with *Terminalia arjuna* Wigh and Arn (Combretaceae) bark extract [30].

Other metallic NPs mediated by bark extracts are: Palladium nanoparticles (PdNPs), with 12.6 nm dimensions, a spherical and quasi-spherical shape, and FCC structure; and copper nanoparticles (CuNPs), with ~20 nm dimensions and a spherical shape. As for the combination of silver and gold nanoparticles, studies showed dimensions of about 15–80 nm and hexagonal, elliptical, and spherical shapes. More details about nanoparticle types, sizes, and shapes, and the phytoconstituents responsible for reduction are revealed in Table 1.

**Table 1:** Synthesis of metallic nanoparticles mediated by the bark of woody plants.

Source of Bark: Scientific Name (Family)—Common Name	NP Type	Size (nm)	Shape	Phytoconstituents Responsible for the Reduction	Reference
*Acacia leucophloea* Roxb. (Fabaceae)—White kabesak	Ag	17–29	Spherical	Aldehyde/ketone, aromatic, azo, and nitro compounds	[20]
*Afzelia quanzensis* Welw. (Fabaceae)—Pod mahogany	Ag	10–80	Spherical	Phytochemical functional groups (carboxyl, amine)	[21]
*Albizia chevalieri* Harms. (Fabaceae)	Ag	~30	Spherical	Alkaloids, terpenoids, flavonoids, and phenols	[31]
*Alstonia scholaris* (L.) R.Br. (Apocynaceae)—Devil’s tree	Ag	50	FCC	-	[32]
*Artocarpus elasticus* Reinw. (Moraceae)—Benda	Ag	19.74 ± 9.70	FCC	Flavonoids, phenols	[33]
*Azadirachta indica* A. Juss (Meliaceae)—Nimtree or Indian lillac	Ag	19.22	Spherical	-	[34,35]
*Berberis lycium* Royle. (Berberidaceae)	Ag	10–100	Spherical	-	[36]
*Butea monosperma* (Lam.) Laum. (Fabaceae)—Flame-of-the-forest	Ag	35	FCC	Carboxylic acid group	[37]
*Cassia fistula* L. (Fabaceae)—Golden tree	Au	55.2–98.4	-	Reducing sugars and terpenoids, secondary metabolites, such as lupeol, β-sitosterol, and hexacosanol	[38]
*Cinnamomum cassia* L. J. Presl (Lauraceae)—Chinese cinnamon	Ag	25–55	Spherical	Phenol, aldehydes, ketones, carboxylic acids, alkyl halides, aromatic groups	[39]
*Cinnamomum zeylanicum* J. Presl (Lauraceae)—True cinnamon	Ag Au	~11.77 ~46.48	Spherical	-	[40]
*Coccinia grandis* L.Voigt (Curcubitaceae)—ivy gourd	Au	20	Spherical	-	[41]
*Cochlospermum religiosum* (L.) Alston (Bixaceae)—Silk-cotton tree	Ag	20–35	Spherical	Carbohydrate, polyphenols, and protein molecules	[42]
*Crataeva nurvala* Buch.-Ham (Capparaceae)—Varuna	Ag	15.2 ± 1.01	spherical	Lupeol, lupenone, hexadecanoic ester, methyl ester	[43]
*Dillenia indica* L. (Dilleniaceae)—Elephant apple	Ag	15–35	Spherical	flavonol, flavonoids, phenolic compounds, stigmasterol, glycosides, and sulfates of flavonoid	[44]
*Diospyros montana* Roxb. (Ebenaceae)—Bombay ebony	Ag	28	--	Amides, phenol, nitrogen, and aromatic compounds	[45,46]
*Elaeodendron croceum* Thunb. DC. (Celastraceae)—Saffron wood	Ag	12.6–41.4	spherical	Amino acids, proteins, polysaccharides, alkaloids, polyphenols, terpenoids or triterpenes, tannins, saponins, and vitamins	[4,47,48,49]
*Eucalyptus globulus* Labill. (Myrtiaceae)—Southern blue gum	Ag Au	21 ± 4 52 ± 16	FCC	Phenolic compounds, particularly galloyl derivatives, glucose and fructose, hydrolyzable tannins	[19]
*Eucommia ulmoides* Oliv. (Eucommiaceae)—Hardy rubber tree	Au	15–40	Spherical FCC	-	[50]
	Pd	12.6	Spherical and quasi-spherical with FCC	Polyphenols, phytosterol, flavonoids, alkaloids, triterpenoids, aminoacids, and proteins	[51]
*Eysenhardtia polystachya* Ort. Sarg. (Fabaceae)—Kidneywood tree	Ag	10–12	Spherical	Arylnaphthalenes, chalcones, flavonoids, and dihydrochalcones	[52]
*Fagus sylvatica* L. (Fagaceae)—Beech	Ag	32	spherical	Tannins and polyphenols	[9,11]
*Ficus benghalensis* var. *krishnae* (Moraceae)—Krishna butter cup	Ag	15–28	Spherical	Phenols, flavonoids, tannins, terpenoids, proteins, alkaloids, saponins, and vitamines	[53,54]
*Ficus benghalensis* (Moraceae)—Banyan tree *Azadirachta indica* A. Juss (Meliaceae)—Nimtree or Indian lilac	Ag	40–50	Spherical	Flavonoids, terpenoids, and phenols	[55]
*Garcinia mangostana* L. (Clusiaceae)—Mangosteen	Ag	12–15	Spherical	Polyphenols	[56]
*Guazuma ulmifolia* Lam. (Malvaceae)—Bay cedar	Ag Au Ag–Au	10–15 20–25 10–20	Spherical	Tannins	[57]
*Holarrhena antidysenterica* L. (Aponycaceae) Wall.—Tellicherry bark or conessi	Ag	32	Spherical	Terpenoids, alkaloids, flavonoids, and phenols	[58]
*Melia azedarach* L. (Meliaceae)—Indian lilac	Ag	30–45	Spherical	Phenolic compounds	[59,60,61]
	Ag Ag–Au	4–30 15–80	Spherical, hexagonal, elliptical	Triterpenoids, flavonoids, glycosides steroids, and carbohydrates	[62]
*Mimusops elengi* L. (Sapotaceae)—Bullet wood	Au	9–14	Spherical	Gallic acid, pinocembrin, quercetin, chlorogenic acid	[14]
*Moringa oleifera* Lam. (Moringaceae)—Moringa	Ag	40	Spherical, pentagon	Terpenoids, flavonoids, and polysaccharides	[26]
*Nerium oleander* L. (Apocynaceae)—Karabi	Au	20–40	Spherical	Flavonoids, steroids, and other secondary metabolites	[63]
*Picea abies* L. (Pinaceae)—Spruce	Ag	44	spherical or rarely polygonal	Catechin, vanillic and gallic acids	[10,64]
*Pinus eldarica* (Pinaceae)—Eldarica pine	Ag	10–40	Spherical	Catechin, taxifolin, procyanidins, and phenolic acids	[65]
*Pongamia pinnata* (L.) Pierre (Fabaceae)—Karum tree	Ag	5–55	Spherical	Phenolic amides, piperine, polysaccharides, and other reducing sugars	[66]
*Prosopis juliflora* Sw.DC. (Fabaceae)—Mesquite	Ag	10–50	Spherical	Flavonoids, alkaloids, and other phenolic compounds	[67]
*Quercus* sp. (Fagaceae)—Oak	Ag	-	-	Tannic acid, glucose, gallic acid	[68]
*Salix alba* L. (Salicaceae)—Willow tree	Au	~15	Spherical	Tannins, alkanoids, flavonoids	[69]
*Saraca indica* L. (Fabaceae)—Asoka tree	Au	15–23	Triangular, polygonal, spherical	Quercetin, epicatechin, catechin, leucopelargoni- din-3-O-p-D-glucoside, gallic acid, leucocyanidin	[28]
*Salvadora persica* L. (Salvadoraceae)—Toothbrush tree	Ag	50	Spherical	Tannins, flavonoids, alkaloids,	[23]
*Shorea roxburghii* D. Don (Dipterocarpaceae)	Ag	4–50	Spherical	Phenolic compounds	[70]
*Stereospermum suaveolens* Roxb. DC (Bignoniaceae)	Ag Au	11.11 12.67	Spherical	Lignans, polyphenols	[71]
*Syzygium alternifolium* (Wt.) Walp (Myrtiaceae)	Ag	4–48	Spherical	Ascorbic acid	[72]
*Syzygium cumini* L. (Myrtiaceae)—Black plum	Ag	20–60	Spherical	Phenols, tannins, alkaloids, glycosides, amino acids, and flavones	[73]
*Syzygium jambos* (L.) Alston (Myrtaceae)	Ag Au	3–10 4–11	Spherical, ellipsoidal	Saccharides and phenolics	[29]
*Terminalia arjuna* Wigh and Arn (Combretaceae)—Arjuna tree	Ag	30–50	Spherical	Polyphenols and proteins	[74]
	Au	3–70	Spherical, triangular FCC	Catechin, gallic acid, ellagic acid	[30]
	Cu	~23	Spherical	Polyphenols (flavonoids), terpenoids, ketones, aldehydes	[75]
	Cu–Ag	~20–30	Spherical	Polyphenols, flavonoids, terpenoids, and reducing sugars	[17]
*Terminalia cuneata* Roth. (Combretaceae)—White murdah	Ag	20–50	Spherical	Hydrolyzable tannins, gallic acid, polyphenols	[76]
*Toxicodendron vernicifluum* (Stokes) F. Barkley (Anacardiaceae)—Chinese Lacquer tree	Ag	2–40	Spherical, oval	Amine, amide, phenolic, and alcoholic aromatics	[27]
*Zizyphus xylopyrus* Retz. Willd (Rhamnaceae)—Kath ber	Ag	60–70	Spherical	-	[77]

Note: Ag—silver nanoparticles; Au—gold nanoparticles; Ag–Au—combination between silver and gold nanoparticles; Cu—copper nanoparticles; Pd—palladium nanoparticles; FCC—face-centered-cubic structure.

## 4. Applications of Metallic NPs Mediated by Plant Bark Extracts

### 4.1. Antioxidant Activity

Phytochemicals present in plants are of great interest. Because of their antioxidant activity, they have beneficial effects on human health and can offer protection against oxidative stress [16]. Parashant et al. [78] revealed that the phenolic compounds present in plant extracts might have a great role in the green synthesis of nanoparticles due to their high antioxidant activity. It was observed that polyphenols are potent antioxidants that can neutralize free radicals because they either donate their electrons or their hydrogen atoms. The generation of free radicals is stopped because of inhibition of the precursors of free radicals or deactivation of active species. Most often, they act as direct radical scavengers of the lipid peroxidation chain reaction (chain breakers). Chain breakers donate an electron to the free radical, making the radical become more stable and interrupting the chain reactions [79,80].

There are many ways to demonstrate the antioxidant activity of certain compounds. One of the well-known methods is measuring the 2,2-Diphenyl-1-picrylhydrazyl (DPPH) radical scavenging activity. This compound is a stable nitrogen-centered free radical and shows a characteristic absorption at 517 nm, whose color changes from violet to yellow upon reduction [81]. Yallapa et al. [75] showed that CuNPs obtained by *Terminalia arjuna* Wight and Arn (Combretaceae) bark extract has antioxidant activity. By donating its electron, it halted the activity of DPPH.

Total reducing power (antioxidant potential) was expressed as ascorbic acid equivalent (AAE) [82]. It was observed that antioxidant activity exhibited by metallic nanoparticles is attributed to polyphenols present in the plant extracts. Silver nanoparticles have more significant antioxidant activity than gold nanoparticles because of their susceptibility to losing electrons [83].

### 4.2. Antibacterial Activity

The mechanism of the antibacterial activity of metallic nanoparticles has been intensely investigated. After binding of NPs on the bacterial cell membrane through electrostatic interactions, the cell wall is disrupted. The intracellular processes, such as DNA, RNA, and protein synthesis, are also affected [84,85,86,87]. The bacteria’s cell membrane is negatively charged, and that may interact with the positively charged metal ion [88].

The synthesized NPs are highly toxic to bacteria when compared to fungus. It was demonstrated that they interact with proteins or possibly phospholipids associated with the proton pump of the bacterial cell wall [89]. That interaction between microorganisms and NPs was commented on by Dibrov et al. [90]. They found that the disruption of the membrane proton gradient could lead to cell death through perturbation of the cellular metabolism mechanisms.

Feng et al. [91] and Matsumura et al. [92] proposed that silver nanoparticles are attached to the cell membrane by sulfur-containing proteins. The AgNPs penetrate inside the bacteria and release silver ions, which interact with the thiol groups of many enzymes. Thus, most of the respiratory chain enzymes are inactivated, leading to the formation of reactive oxygen species (ROS), which causes the self-destruction of the bacterial cell [54].

Ali et al. [43] found that AgNPs obtained by *Crataeva nurvala* Buch.-Ham (Capparaceae) bark extract were internalized inside the *Pseudomonas aeruginosa* bacterial cells, leading to cell death. The metallic nanoparticles can restrict the bacterial colonization and also inhibit the biofilm formation produced by bacterial quorum sensing activity (quorum sensing virulence factors responsible for multidrug resistance), a fact that is can be evidenced by confocal laser scanning microscopes.

Many studies have looked at the NP effects against *Escherichia coli* and *Staphylococcus aureus*, the models for Gram-negative and Gram-positive bacteria. Moreover, they have been studied because of the contrast between the cell wall structures of the two bacterial cell types. The cell walls of Gram-positive bacteria have a rigid layer of liner polysaccharide that does not exist in Gram-negative bacteria [93]. However, the principal component of the cell walls of both Gram-positive and Gram-negative bacteria is a peptidoglycan consisting of linear polysaccharide chains crosslinked by short peptides [94]. The antibacterial activity was also revealed by Miri et al. [23]. The AgNPs obtained with *Salvadora persica* L. (Salvadoraceae) in *Escherichia coli* and *Staphylococcus aureus* showed inhibition zones of 14 and 12 mm, respectively. The minimum inhibitory concentration (MIC) results against studied bacteria were 100 and 400 μg/mL for *Escherichia coli* and *Staphylococcus aureus*, respectively. As an observation, in this study, it was found that the synthesized AgNPs not only inhibited the growth of *Escherichia coli* but also had a bactericidal effect.

The study by Arya et al. [67] showed that *Escherichia coli* and *Pseudomonas aeruginosa* were susceptible to *Prosopis juliflora* Sw.DC. (Fabaceae)-obtained AgNPs. The small nanoparticles (10–55 nm) could easily enter into the bacterial cells and disturb their usual organization. This study revealed that the power of synthesized AgNPs is dose-dependent, and that they can also overcome the multidrug resistance problem.

Francis et al. [71] evaluated the antimicrobial effect of AgNPs and AuNPs obtained from *Stereospermum suaveolens* Roxb. DC (Bignoniaceae). They were evaluated using the disc diffusion method by adding 50 µL of the samples. It was observed that the effect of inhibition was more pronounced in the case of Gram-negative bacteria than Gram-positive bacteria because of their cell wall differences. Silver nanoparticles possessed a higher degree of microbial inhibition than the gold nanoparticles. This higher degree is because silver is a soft acid that preferably binds sulfur and phosphorus fragments in the cell, leading to DNA damage and cell death [25,93,95].

A study by Yallapa et al. [17] presented the antibacterial properties of AgNPs and CuNPs obtained with *Terminalia arjuna* Wight and Arn (Combretaceae) bark extract against Gram-positive and Gram-negative bacteria. The structural changes induced in the bacterial cell wall and the nuclear membrane led to cell death. All this was due to the extremely reactive action of silver and copper nanoparticles and because of the ease of binding to tissue proteins [96]. More metallic nanoparticles obtained with bark extract constituents and their antimicrobial activity are presented in Table 2.

### 4.3. Anticancer Activity

With a wide range of applications, including medical and therapeutic assistance, nanoparticles have been reported as having excellent anticancer activity. This is due to the involvement in the selective interruption of the mitochondrial respiratory chain, which results in the production of ROS. ROS species induce the expression of genes associated with DNA disruption and produces apoptosis of tumor cells [97]. Considering the size of metallic nanoparticles and sample concentration, it was observed that silver nanoparticles induce damage to a cancer cell in a dose- and size-dependent manner [98]. Higher doses of smaller size particles create more cytotoxic effects on the tumoral tissue. The tumoral cells present a characteristic acid pH, and metallic nanoparticles have an affinity for this specific environment. Because of this reason it is believed that treatment with these nanoparticles has the advantage of targeting the tumoral cells and minimizing side effects on the healthy cells [99,100].

### 4.4. Other Activities

One of the applications of nanoparticles is the deterioration of pollutants as nitroaromatic compounds and dyes. Because of the hazardous and toxic effect of the majority of organic compounds on humans, there are many studies about their removal from nature. Many industrial compounds, such as pharmaceuticals, pigments, dyes, plastics, pesticides, fungicides, explosive, and industrial solvents, contain these pollutant solvents [101]. The U.S. Environmental Protection Agency concluded that of all the studied nitroaromatic compounds, 4-nitrophenol is a priority pollutant because it is very stable and resistant to biodegradation [101,102].

To study the catalytic activity of the synthesized gold nanoparticles using the extract of *Mimusops elengi* L. (Sapotaceae), two model reactions were carried out, both monitored by UV-visible spectroscopy: the reduction of 3-nitrophenol to 3-aminophenol and 4-nitrophenol to 4-aminophenol [14]. The catalytic activity of AuNPs was demonstrated. The reduction was observed over 300 s via UV spectroscopy, indicating completion of the reduction. Interestingly, with the addition of a double amount of AuNPs, the reaction was completed much faster (in 30 s), and the catalytic rate constant could not be calculated for this reduction reaction. The excellent catalytic activity was demonstrated in the reduction reaction of 4-nitrophenol in the same way [14].

One of the most harmful actions of dyes is that they form a foam on the surface of water. The foam blocks the diffusion of light and oxygen, with consequences on the biological phenomena of the aquatic medium. Another effect of these chemicals and dyes is that they are very poisonous and strongly oncogenic [103].

Because of the undesirable effect of the insecticides on human health and other non-targeted organisms, Velayutham et al. [104] noted the larvicidal effect of aqueous crude bark extract and synthesized AgNPs of *Ficus racemosa* L. (Moraceae). The highest mortality was found in synthesized AgNPs against the larvae *Culex quinquefasciatus* and *Culex gelidus* at the concentration of 25 mg/L. All tested samples showed that lethal effects and mortality were positively dose-dependent.

Daisy and Sapriya [38] concluded that AuNPs mediated by *Cassia fistula* bark extract have potential anti-diabetic properties. These NPs showed a glucose reduction in blood serum concentration, induced favorable changes in body weight, improved transaminase activity, achieved a better lipid profile, and reversed renal dysfunction to a greater extent.

Garcia Campoy et al. [52] found that AuNPs obtained with *Eysenhardtia polystachya* Ort. Sarg. (Fabaceae) bark extract promoted pancreatic β-cell survival, insulin secretion, and enhanced hyperglycemia and hyperlipidemia in glucose-induced diabetic zebrafish. These studies show promising results, but other studies may be needed to develop new methods for practical disease treatment. Other applications of metallic nanoparticles can be found in Table 2.

**Table 2:** Applications of nanoparticles mediated by the bark of woody plants.

Source of Bark: Scientific Name (Family)—Common Name	NP Type	Activity	Reference
*Acacia leucophloea* Roxb. (Fabaceae)—White kabesak	Ag	Antibacterial activity against the common pathogens, such as *Staphylococcus aureus*, *Bacillus cereus*, *Listeria monocytogenes*, and *Shigella flexneri*	[20]
*Afzelia quanzensis* Welw. (Fabaceae)—pod mahogany	Ag	Antibacterial against *Escherichia coli*, *Staphylococcus aureus*	[21]
*Albizia chevalieri* Harms. (Fabaceae)	Ag	Antibacterial against *Escherichia coli*, *Staphylococcus aureus*; Anticancer against *MDA-MB231*, *MCF-7* breast cancer cell line, and *HepG2* liver cancer cell line	[31]
*Alstonia scholaris* (L.) R.Br. (Apocynaceae)—Devil’s tree	Ag	Antimicrobial activity against fungal species, and Gram-positive and Gram-negative bacteria	[32]
*Azadirachta indica* A. Juss (Meliaceae)—Nimtree or Indian lillac	Ag	Larvicidal against the larvae, pupae, and adults of malaria vector *Anopheles stephensi* and filariasis vector *Culex quinquefasciatus*	[35]
*Berberis lycium Royle.* (Berberidaceae)	Ag	Antimicrobial activities against both Gram-negative bacteria (*Escherichia coli*, *Klebseilla pneumoniae*, *Pseudomonas aeruginosa*) and Gram-positive bacteria (*Staphylococcus aureus and Bacillus subtilis*)	[36]
*Butea monosperma* (Lam.) Laum. (Fabaceae)—Flame-of-the-forest	Ag	Antibacterial activity against Gram-positive (*Bacillus subtilis*) and Gram-negative (*Escherichia coli*)	[37]
*Cassia fistula* L. (Fabaceae)—Golden tree	Au	Antidiabetic: reduces serum blood glucose concentrations, induces favorable changes in body weight, improves transaminase activity, achieves a better lipid profile, and reverses renal dysfunction to a greater extent	[38]
*Cinnamomum cassia* L. J. Presl (Lauraceae)—Chinese cinnamon	Ag	Non-toxic against *Vero* cells Antiviral activity against *H7N3 influenza virus*	[39]
*Cinnamomum zeylanicum* J.Presl (Lauraceae)—True cinnamon	Ag Au	Antibacterial: EC50 value of 11 ± 1.72 mg/L against *Escherichia coli* BL-21 strain	[40]
*Coccinia grandis* L.Voigt (Curcubitaceae)—ivy gourd	Au	Increasing biocompatibility and bioavailability of N-acetylcysteine drug molecule that is used for cataract treatment, which was successfully encapsulated into AuNPs	[41]
*Cochlospermum religiosum* (L.) Alston (Bixaceae)—Silk-cotton tree	Ag	Antibacterial activity against *Staphylococcus aureus*, followed by *Pseudomonas, Escherichia coli, Bacillus*, and lowest activity toward *Proteus* Antifungal against *Aspergillus flavus*, followed by *Rhizopus*, *Fusarium*, and *Curvularia*	[42]
*Crataeva nurvala* Buch.-Ham (Capparaceae)—Varuna	Ag	Antibiofilm properties in *Pseudomonas aeruginosa*	[43]
*Dillenia indica* L. (Dilleniaceae)—Elephant apple	Ag	Catalytic degradation of 4-Nitrophenol, methylene blue; radical scavenging activity	[44]
*Diospyros montana* Roxb. (Ebenaceae)—Bombay ebony	Ag	Antibacterial activity in vitro against Gram-positive (*Bacillus subtilis*, *Staphylococcus aureus*) and Gram-negative (*Escherichia coli*, *Klebsiella aerogenes*); antioxidant activity	[45,46]
*Elaeodendron croceum* Thunb. DC. (Celastraceae)—Saffron wood	Ag	Cytotoxic activity against the MDA-MB-231 cell line	[47]
*Eucommia ulmoides* Oliv. (Eucommiaceae)—Hardy rubber tree	Au	Excellent performance for the catalytic decoloration of reactive yellow 179 and Congo red by NaBH4 in aqueous solution	[50]
	Pd	Catalytic activity for the electro-catalytic oxidation of hydrazine and the catalytic reducing degradation of p-Aminoazobenzene	[51]
*Eysenhardtia polystachya* Ort. Sarg. (Fabaceae)—Kidneywood tree	Ag	Promote pancreatic β-cell survival, insulin secretion Enhances hyperglycemia and hyperlipidemia in glucose-induced diabetic zebrafish	[52]
*Fagus sylvatica L.* (Fagaceae)—Beech	Ag	Antioxidant and antibacterial against Gram-positive and Gram-negative bacteria	[9]
*Ficus benghalensis* var. *krishnae* (Moraceae)—Krishna butter cup	Ag	Antimicrobial activity against *Staphylococcus aureus* (ATCC 29122), *Escherichia coli* (MTCC 45) and *Salmonella typhimurium* (MTCC 98) Cytotoxicity on ovarian cancer cell lines (*SKOV-3 cells*)	[53,54]
*Ficus benghalensis* (Moraceae)—Banyan trees *Azadirachta indica* A. Juss (Meliaceae)—Nimtree or Indian lilac	Ag	Antibacterial against *Escherichia coli*, *Pseudomonas aeruginosa*, *Bacillus subtilis*, and *Vibrio cholerae* Antiproliferative against *MG-63* ostheosarcoma cell line	[55]
*Ficus racemosa* L. (Moraceae)—Indian fig tree	Ag	The larvicidal activity results showed the highest mortality in synthesized AgNPs compared with the aqueous bark extract of *F. racemosa*	[104]
*Garcinia mangostana* L. (Clusiaceae)—Mangosteen	Ag	Anticancer activity in lung cancer cells (A549)	[56]
*Guazuma ulmifolia* Lam. (Malvaceae)—Bay cedar	Ag–Au	Anticancer activity against HeLa cells Antibacterial and antifungal activity Catalityc activity against Congo red and 4-nitrophenol	[57]
*Holarrhena antidysenterica* L. (Aponycaceae) Wall.—Tellicherry bark or conessi	Ag	Larvicidal activity against the larvae of *Aedes aegypti* and *Culex quinquefasciatus*	[58]
*Melia azedarach* L. (Meliaceae)—Indian lilac	Ag	Antibacterial against *Esherichia coli, Klebsiella pneumoniae*	[59]
	Ag Ag–Au	Antibacterial against *Bacillus cereus*, *Cronobacter sakazakii*, *Salmonella enterica*, *Escherichia coli*, *Listeria monocytogenes*, *Candida albicans*	[62]
*Mimusops elengi* L. (Sapotaceae)—Bullet wood	Au	Efficient catalyst for the reduction of 3-nitrophenol and 4-nitrophenol to their corresponding aminophenols in water at room temperature	[14]
*Moringa oleifera* Lam. (Moringaceae)—Moringa	Ag	Anticancer activity against HeLa cell type (human cervical carcinoma)	[26]
*Nerium oleander* L. (Apocynaceae)—Karabi	Au	In vitro anticancer activity of the stabilized AuNPs on MCF-7 cell lines; catalytic activities demonstrated for borohydride reduction of 3- and 4-nitrophenols	[60]
*Picea abies* L. (Pinaceae)—Spruce	Ag	Antioxidant and Antibacterial against Gram-positive and Gram-negative bacteria	[10]
*Pongamia pinnata* (L.) Pierre (Fabaceae)—Karum tree	Ag	Antibacterial activity against *Klebsiella planticola* and *Staphylococcus aureus*	[66]
*Prosopis juliflora* Sw.DC. (Fabaceae)—Mesquite	Ag	Antibacterial activity against *Escherichia coli* and *Pseudomonas Aeruginosa*; Anticancer activity against *A549* cells (adenocarcinomic human alveolar basal epithelial cells); photocatalytic degradation of 4-nitrophenol	[67]
*Quercus* sp. (Fagaceae)—Oak	Ag	Antibacterial effect against *Staphylococcus aureus ATCC 25923*, *Listeria monocytogenes ATCC 19111*, *Bacillus cereus ATCC 11778*, *Escherichia coli ATCC 25922*, *Salmonella enterica* subsp. *enterica Serovar typhimurium ATCC 13076* reference strains —cultures were isolated from food products	[68]
*Salix alba* L. (Salicaceae)—Willow tree	Au	Used in colorimetric detection of cysteine	[69]
*Saraca indica* L. (Fabaceae)—Asoka tree	Au	Catalyst for the reduction of 4-nitrophenol to 4-aminophenol	[28]
*Salvadora persica* L. (Salvadoraceae)—Toothbrush tree	Ag	Antibacterial activity against *Escherichia coli* and *Staphylococcus aureus*	[23]
*Stereospermum suaveolens* Roxb. DC (Bignoniaceae)	Ag Au	Anticancer activity against lung carcinoma cell lines A549 Antibacterial against *Escherichia coli*, *Staphylococcus aureus*, *Pseudomonas aeruginosa*, *Bacillus subtilis* Antifungal against *Aspergillus flavus*, *Aspergillus nidulans*	[71]
*Syzygium alternifolium* (Wt.) Walp (Myrtiaceae)	Ag	Antibacterial activity against *Salmonella typhimurium*, *Proteus vulgaris*, *Klebsiella pneumoniae*, *Escherichia coli*, *Pseudomonas aeruginosa*, *Staphylococcus aureus*, and *Bacillus subtilis* Antifungal: highest inhibition zones are observed in *Aspergillus flavus* followed by *Penicillium chrysogenum*, *Trichoderma harzianum*, *Alternaria solani*, and *Aspergillus Niger*	[72]
*Syzygium cumini* L.(Myrtiaceae)—Black plum	Ag	Antibacterial against *Escherichia coli*, *Staphylococcus aureus*, *Bacillus licheniformis*	[73]
*Syzygium jambos* (L.) Alston (Myrtaceae)	Ag Au	Antiplasmodial effect (AgNPs > AuNPs) against both chloroquine sensitive (3D7) and resistant (Dd2) strain of *Plasmodium falciparum*	[29]
*Terminalia arjuna* Wigh and Arn (Combretaceae)—Arjuna tree	Ag	Antibacterial against *Escherichia coli*	[74]
	Au	Reducing and capping agent; acetylcholinesterase and Butyrylcholinesterase inhibitory activities; excellent free radical scavenging and metal chelating activity, suitable for Alzheimer’s disease therapy.	[30]
	Cu	Antioxidant properties; Antibacterial activity against *Escherichia coli* and *Staphylococcus aureus* and less effective against both *Pseudomonas aeruginosa* and *Salmonella typhium*; Effective against *Candida albicans*, *Trichophyton rubrum*, *Chrisosporium indicum*	[75]
	Cu–Ag	Cytotoxic effect of biohybrid nanomaterials on different cell lines, MDA-MB-231 (poorly differentiated triple-negative breast cancer), HeLa (cervical cancer cells), SiHa (squamous cell carcinoma), and He-G2 (liver cancer cells), and non-toxic against Vero (normal epithelial cells); antibacterial activity against bacterial strains *Escherichia coli*, *Staphylococcus aureus*	[17]
*Terminalia cuneata* Roth. (Combretaceae)—White murdah	Ag	Catalytic activity in the reduction of direct yellow-12	[76]
*Toxicodendron vernicifluum* (Stokes) F.Barkley (Anacardiaceae)—Chinese Lacquer tree	Ag	Anticancer activity in human lung cancer A549 cells Antibacterial activity against STEC (*Shiga Toxina Escherichia Coli*) and *Helicobacter pylori*	[27]
*Zizyphus xylopyrus* Retz. Willd (Rhamnaceae)—Kath ber	Ag	Antimicrobial agents in water purification systems	[77]

Ag—silver nanoparticles; Au—gold nanoparticles; Ag–Au—combination of silver and gold nanoparticles; Cu—copper nanoparticles; Pd—palladium nanoparticles.

## 5. Conclusions

The bark of woody vascular plants can be a source of phytoconstituents responsible for the reduction of metallic ions in nanoparticle synthesis. In this review, the presented metallic NPs mediated by bark extracts are rich in phytoconstituents responsible for the reduction of metal salts. The synthesis of NPs using crude plant bark extracts and purified compounds are novel substrates for industrial production.

In the future, plant bark has a wide potential for the synthesis of NPs in health care and commercial products. Implementing green synthesis methods with proven advantages has great potential. The yield of synthesized nanoparticles remains to be elucidated, and the synthesis parameters still require optimization. Furthermore, the lack of knowledge about chemical components responsible for the synthesis and stabilization process of NPs remains a challenge for researchers. It is important to understand how bioactive groups attach to the surface of NPs and which bioactive groups are involved in order to mediate NPs with higher efficacy. However, issues relating to the biomedical applications of NPs in vivo need to be developed. At the same time, considerable research on the biocompatibility and bioavailability of NPs is needed.
